# Recyclable and Stable Porphyrin‐Based Self‐Assemblies by Electrostatic Force for Efficient Photocatalytic Organic Transformation

**DOI:** 10.1002/advs.202308469

**Published:** 2024-03-09

**Authors:** Bin Cai, Ping Huang, Yuan Fang, Haining Tian

**Affiliations:** ^1^ Department of Chemistry‐Ångström Lab Uppsala University Box 523 Uppsala SE 751 20 Sweden; ^2^ Department of Chemistry KTH Royal Institute of Technology Teknikringen 30–36 Stockholm SE 100 44 Sweden

**Keywords:** electrostatic assemblies, photoredox catalysis, porphyrin, superoxide, thioanisole

## Abstract

Development of efficient, stable, and recyclable photocatalysts for organic synthesis is vital for transformation of traditional thermal organic chemistry into green sustainable organic chemistry. In this work, the study reports an electrostatic approach to assemble meso‐tetra (4‐sulfonate phenyl) porphyrin (TPPS)tetra (4‐sulfonate phenyl) porphyrin (TPPS) as a donor and benzyl viologen (BV) as an acceptor into stable and recyclable photocatalyst for an efficient organic transformation reaction – aryl sulfide oxidation. By use of the electrostatic TPPS‐BV photocatalysts, 0.1 mmol aryl sulfide with electron‐donating group can be completely transformed into aryl sulfoxide in 60 min without overoxidation into sulfone, rendering near 100% yield and selectivity. The photocatalyst can be recycled up to 95% when 10 mg amount is used. Mechanistic study reveals that efficient charge separation between TPPS and BV results in sufficient formation of superoxide which further reacts with the oxidized sulfide by the photocatalyst to produce the sulfoxide. This mechanistic pathway differs significantly from the previously proposed singlet oxygen‐dominated process in homogeneous TPPS photocatalysis.

## Graphical abstract

A kind of porphyrin‐viologen‐based self‐assembly by electrostatic force has been prepared. It is found that the efficient photo‐induced electron transfer from the excited tetra (4‐sulfonate) porphyrin (TPPS) donor to the benzyl viologen (BV) acceptor exists in the TPPS‐BV self‐assembly, which is beneficial to sulfide oxidation when acting as the photocatalyst. Correspondingly, TPPS‐BV exhibits a remarkable fourfold increase in catalytic efficiency compared to TPPS‐EDA when changing BV into ethylene diammonium (EDA).

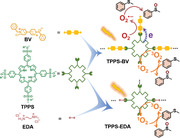

## 1 Introduction

Organic synthesis achieved with the photocatalysts under the light‐driven catalytic cycle has drawn great interest because of their easy operation and eco‐friendly benefits.^[^ ^1^ ^]^ Upon light illumination, the excited photocatalyst can either contribute electrons to the substrate, functioning as a reduction reagent,^[^ ^2^ ^]^ or receive electron from the substrate, serving as an oxidation reagent.^[^ ^3^ ^]^ According to the dispersibility in macroscale, the commonly used photocatalysts can be categorized into two main groups: homogeneous and heterogeneous.^[^ ^4^ ^]^ Homogeneous photocatalysts offer the advantage of efficient mixing with reactants, therefore enhancing the reaction contact area and accelerating reaction rates. However, they encounter challenges related to efficient recyclability.^[^ ^5^ ^]^ On the other hand, heterogeneous photocatalysts are recognized for their straightforward recycling processes, they often suffer from reduced efficiency in mixing with reactants due to their bulk nature. Therefore, the development of efficient and easily recyclable photocatalysts for specific organic reactions with high yield and selectivity is desirable. Moreover, organic photocatalysts have advantages over inorganic photocatalysts due to their flexible structure modification,^[^ ^6^ ^]^ tunable energy alignment,^[^ ^7^ ^]^ convenient to be purified, and abundant building blocks.^[^ ^8^ ^]^ The porphyrin ring, characterized by a 26‐*π* electron delocalized aromatic system, has been reported to be a kind of efficient and stable photocatalyst owing to its unique planarity structure and electronic properties.^[^ ^9^ ^]^ These qualities make porphyrin compounds significantly valuable in photocatalytic organic synthesis. They have found extensive application in various reactions such as H_2_O_2_ production reaction,^[^ ^10^ ^]^ oxidation reaction,^[^ ^11^ ^]^ arylation reaction,^[^ ^12^ ^]^ dehalogenation reaction,^[^ ^13^ ^]^ etc. These reactions involved porphyrin in diverse forms, including metal‐organic framework (MOF), covalent‐organic framework (COF), modified porphyrin complex, etc.^[^ ^14^ ^]^

To date, many organic reactions have been driven by organic photocatalysts.^[^ ^15^ ^]^ Among these, thioaniosle oxidation has been regarded as an important reaction due to the key role of its sulfoxide product in the pharmaceutical industry owing to its biological activity, and its utility as a common intermediate in organic synthesis.^[^ ^16^ ^]^ Synthesis of sulfoxide by use of organic photocatalysts has been well demonstrated.^[^ ^17^ ^]^ Among organic photocatalysts for thioanisole oxidation, porphyrin‐based photocatalysts exhibited great promise owing to their high yield of triplet state, which is beneficial for the production of the singlet oxygen through energy transfer, as well as the potential to perform the electron transfer.^[^ ^18^ ^]^ However, previously reported porphyrin‐based photocatalysts have often faced challenges, such as poor recyclability or intricate synthesis procedures.^[^ ^19^ ^]^ To address these issues, in this work, we designed two self‐assembled photocatalysts based on electrostatic interaction between meso‐tetra (4‐sulfonate phenyl) porphyrin (TPPS) and benzyl viologen (BV) or ethylene diammonium (EDA) for sulfide oxidation into sulfoxide. TPPS‐BV assembly showed much higher sulfoxide production performance as compared to TPPS‐EDA assembly. Efficient charge separation between TPPS and BV has been demonstrated, giving effective production of the superoxide which is responsible for the enhanced photocatalytic performance. Notably, after the initial reaction and subsequent washing, a remarkable 95% of TPPS‐BV could still be conveniently recycled, highlighting its robust recyclability.

## 2 Results and Discussion

**Figure 1:**
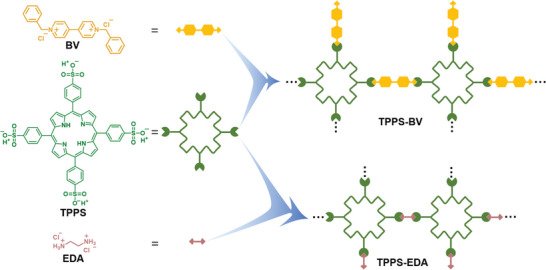
Chemical structures of BV, TPPS, and EDA, and demonstrations of electrostatic self‐assemblies of TPPS‐BV and TPPS‐EDA.

**Figure 2:**
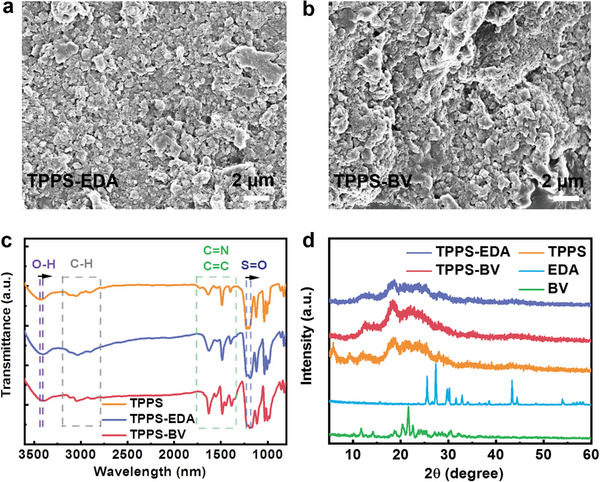
SEM images of a) TPPS‐EDA self‐assembly, and b) TPPS‐BV self‐assembly. c) FT‐IR spectra of the TPPS, TPPS‐EDA self‐assembly, and TPPS‐BV self‐assembly. d) XRD patterns of the BV, EDA, TPPS, TPPS‐EDA, and TPPS‐BV.

**Figure 3:**
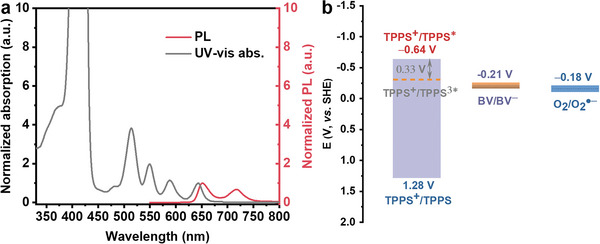
a) UV–vis absorption spectrum and PL emission spectrum of the TPPS measured in DMF. b) Energy level alignment of oxidation potential of ground state TPPS and excited state TPPS, reduction potential of BV and O_2_.

As depicted in **Figure** **1**, BV and EDA (Synthesized found in Figure S1, Supporting Information) represent two distinct types of ammonium cations, while TPPS functions as a sulfonic anion photosensitizer. Upon electrostatic interaction between TPPS and BV or EDA, self‐assemblies were formed and precipitated from the solution, denoted as TPPS‐BV and TPPS‐EDA, respectively. Further elaboration on the procedure for fabricating the self‐assemblies can be found in the Supporting Information (Figure S2, Supporting Information). Scanning electron microscopy (SEM) reveals that both TPPS‐BV and TPPS‐EDA self‐assemblies consist of aggregated small nanoparticles (**Figure** **2**a,b; Figure S3, Supporting Information) and also show mesoporous structures. From the Fourier‐transform infrared spectroscopy (FTIR) spectra, upon their incorporation into the self‐assemblies, the original O─H bond at 3434 cm^−1^ and S═O bond at 1225 cm^−1^ in TPPS exhibited a redshift of 30 and 55 cm^−1^, respectively, indicating the decrease of original bond strength due to electrostatic interactions with another compound (Figure 2c).^[^ ^20^ ^]^ Magic‐angle spinning (MAS) solid‐state ^13^C‐NMR measurements of the TPPS‐BV and TPPS‐EDA also confirm the successful building of the self‐assemblies (Figure S4, Supporting Information). In TPPS‐BV assembly, electrons transfer from the excited TPPS to the ground state BV in TPPS‐BV is thermodynamically feasible as indicated in the potential diagram obtained from electrochemistry and *E*_0‐0_ (values can be found in Supporting Information, **Figure** **3**b; Figure S5 and Table S1, Supporting Information). In contrast, TPPS‐EDA assembly, serving as a control group, is absent of any charge separation process between TPPS and EDA. UV–vis absorption spectra, PL emission spectra, and electrochemical properties of the TPPS‐BV and TPPS‐EDA self‐assemblies were also measured and provided (Figures S6–S8, Supporting Information). The presence of four characteristic absorption peaks within the TPPS Q‐band confirms its metal‐free characteristics (Figure 3a; Figure S9, Supporting Information).^[^ ^21^ ^]^ Power X‐ray diffraction (XRD) analysis indicates that the distinct peaks observed in the original well‐ordered packing of BV and EDA have disappeared upon assembly into TPPS‐BV and TPPS‐EDA, suggesting that TPPS‐BV and TPPS‐EDA possess amorphous structures (Figure 2d).

**Figure 4:**
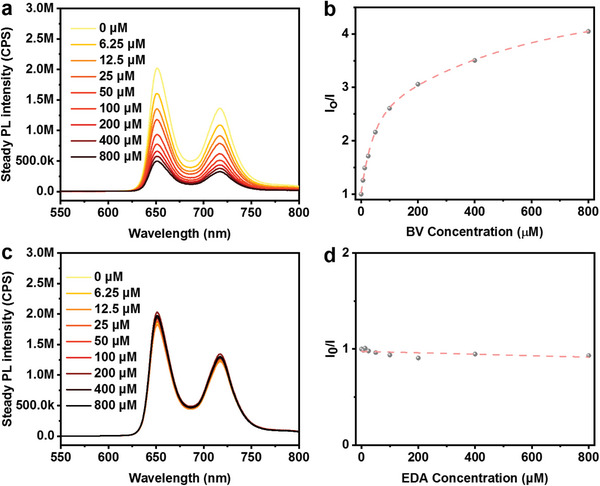
a,c) Steady‐state PL emission quenching of TPPS (40 µm in DMSO) by adding different ratio of BV and EDA, respectively; b,d) Stern–Volmer plot of the TPPS PL intensity quenched by BV and EDA monitored at 650 nm, respectively.

**Figure 5:**
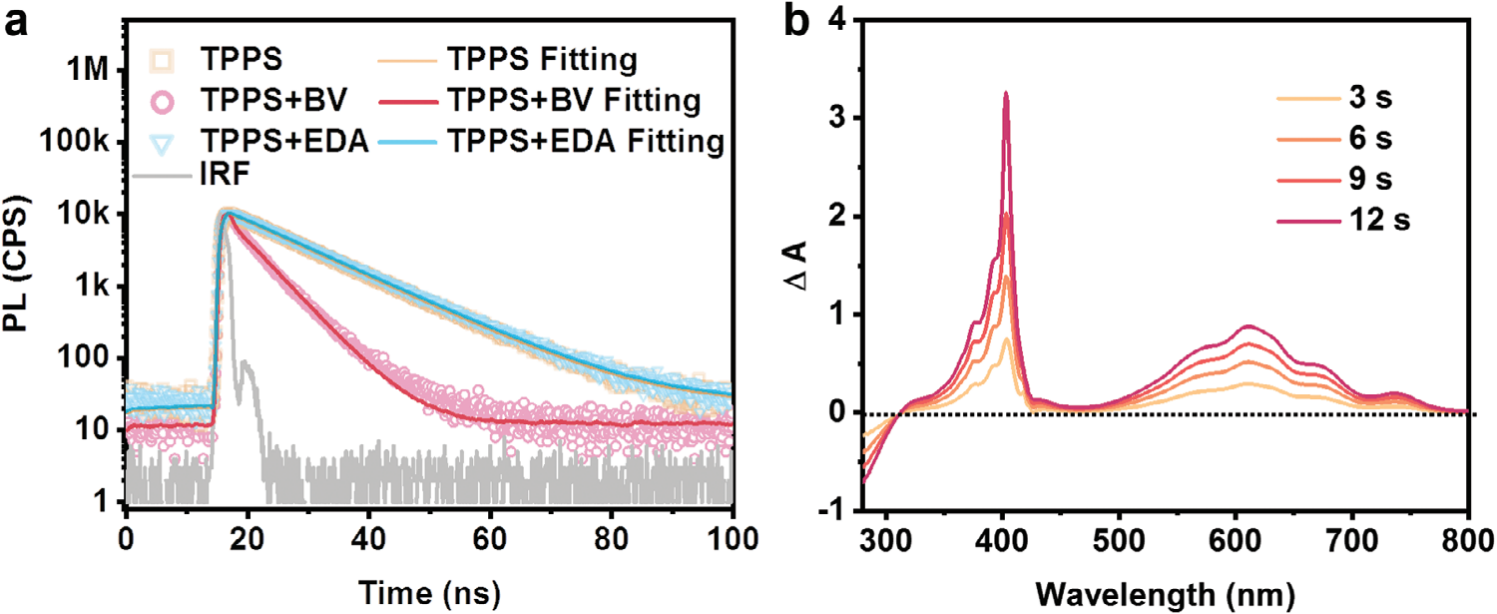
a) TCSPC measurements of TPPS (yellow square), TPPS with BV (pink circle), TPPS with EDA (blue triangle), and the solid lines are corresponding fitting curves, 40 µm TPPS in DMSO, adding 400 µm quencher; b) Differential UV–vis absorption spectra of TPPS added with BV under different illumination time, TEOA added as the electron donor.

In addition to energy alignment assessment, an investigation of charge separation between TPPS and BV was conducted through steady‐state photoluminescence (PL) quenching experiments and time‐correlated single photon counting (TCSPC). To mitigate the prompt precipitation attributed to electrostatic interactions between the TPPS anion and the ammonium cation, dimethyl sulfoxide (DMSO) was chosen as the solvent owing to its good dissolving capacity to avoid precipitation during all the measured concentrations. Notably, with the increase of the BV ratio to TPPS, the PL intensity of TPPS exhibited attenuation gradually, implying the occurrence of an oxidative quenching process (**Figure** **4**a). This is in accordance with TCSPC measurements, where TPPS lifetime is conspicuously diminished from 11.2 to 4.8 ns by adding BV (fitting data in Table S2, Supporting Information), signifying the occurrence of electron transfer from the excited TPPS to the BV moiety (**Figure** **5**a). Furthermore, the interaction between TPPS and BV at relatively elevated BV concentrations was observed from the Stern–Volmer plot, judging from non‐linear characteristics inherent with a saturated value in the plot (Figure 4b).^[^ ^22^ ^]^ Specifically, when the BV concentration attained a sufficiently elevated level, a BV shell encompassing the TPPS was engendered through Coulombic attraction, which inhibits further acceptance of electrons by the additional BV from the excited TPPS, therefore forming the non‐linear characteristic plot. In contrast, the addition of EDA yielded almost no change of PL quenching on TPPS, indicating the absence of charge transfer between the excited TPPS and EDA (Figure 4c,d). Moreover, the PL lifetime of TPPS was not quenched by adding EDA (from 11.2 to 11.4 ns) as shown in Figure 5a.

Elucidation of the light‐induced electron transfer process from TPPS to BV was further illustrated by an obvious observation of characteristic absorption of reduced viologen peaks (405 and 610 nm) upon light illumination of the TPPS and BV mixture, with triethanolamine (TOEA) as the sacrificial electron donor (Figure 5b). Upon light absorption, excited TPPS could transfer electrons to BV initially, as proved by TCSPC (Figure S10, Supporting Information), producing reduced BV, while TOEA facilitates the regeneration of the TPPS ground state through electron transfer to the oxidized TPPS. This process results in the accumulation of the reduced BV with an increase in absorbance at 405 and 610 nm, thereby providing direct evidence of the electron transfer from TPPS to BV.^[^ ^18^, ^23^ ^]^

**Figure 6:**
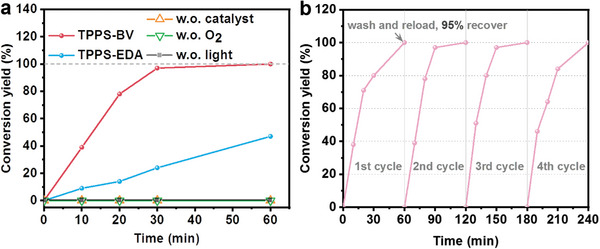
a) Photo‐oxidation of thioanisole under different conditions: “light+O_2_+TPPS‐BV” (red line), “light+O_2_+TPPS‐EDA” (blue line), “light+O_2_” (yellow line), “light+TPPS‐BV” (green line), “O_2_+TPPS‐BV” (grey line) with 2 mL 50 mm thioanisole added with 10 mg photocatslyst; b) Recycle experiment of photo‐oxidation of thioanisole with TPPS‐BV as the catalyst, reaction recycled through centrifuge at the end of reaction, wash with methanol and reload 2 mL 50 mm thioanisole.

**Figure 7:**
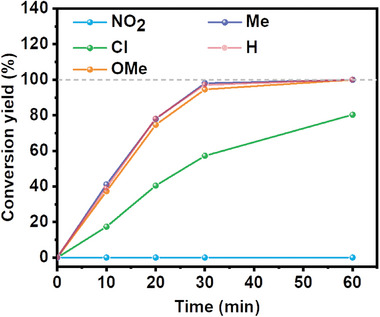
Photocatalytic sulfide oxidation activity of the TPPS‐BV on substrates with different electron‐donating and electron‐withdrawing ability.

**Scheme 1:**
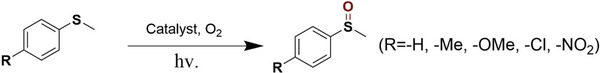
Photocatalytic oxidation sulfide into sulfoxide reaction.

Photo‐oxidation reaction of thioanisole, as the representative substrate, was first conducted to evaluate the photocatalytic activity of as‐prepared self‐assemblies with or without charge separation ability. The photocatalytic reaction was conducted using two LED lamps (Zenaro brand, 17 W, emitting spectrum 420–750 nm), with each lamp possessing an intensity of 50 mW cm^−2^ (Figure S11, Supporting Information). Briefly, 10 mg photocatalyst was added into 2 mL 50 mm sulfide/MeOH in a sealed vial, followed by light illuminating after purging with O_2_. As shown in **Figure** **6**a and Figure S12 (Supporting Information), heterogeneous TPPS‐BV exhibits a comparable photocatalytic efficiency to that of TPPS, while TPPS acts as a homogeneous catalyst in the reaction (images of homogeneous TPPS and heterogeneous self‐assemblies are shown in Figure S13, Supporting Information). By comparison, TPPS‐BV showed catalytic activity exceeding that of TPPS‐EDA by more than four folds, signifying the advantageous impact of the improvement of charge separation in the photocatalyst on the overall photocatalytic performance. The external quantum efficiency of the champion system of TPPS‐BV reached ≈2.5% at 520 nm (Figure S14, Supporting Information). The effect of the molar ratio of TPPS to BV (or EDA) on the photocatalytic performance of TPPS‐BV and TPPS‐EDA has also been conducted and provided in Figures S15 and S16 (Supporting Information). Despite sulfide's tendency for facile over‐oxidation to sulfone as reported by other studies,^[^ ^19^, ^24^ ^]^ no sulfone products were detected even when the reaction time was extended to 150 min (>2.5 times longer than the reaction completion time), indicating the remarkable selectivity toward sulfide oxidation by the TPPS‐BV assembly (Figure S17, Supporting Information). When in the absence of either photocatalyst, O_2_, or light, no methyl phenyl sulfoxide product was obtained, conclusively attributing the reaction to be a light‐driven photocatalytic process (Figure 6a). Upon completion of the reaction, TPPS‐BV was isolated through centrifugation and subsequently washed with methanol. A fresh substrate with solvent was then refilled into the vial to reinitiate a new cycle of photooxidation reaction. There was no obvious decline in photocatalytic activity observed throughout 4 successive recycling experiments, emphasizing the remarkable recyclability of TPPS‐BV photocatalyst (Figure 6b). Notably, 95 wt.% of TPPS‐BV could be successfully recovered following the first cycle reaction even with 10 mg TPPS‐BV used. The tiny loss is probably from the washing and transporting procedure (Figure S18, Supporting Information). Furthermore, TPPS‐BV kept stable during the photocatalytic reaction judging from the negligible changes in powder XRD, FT‐IR, and liquid ^1^H‐NMR spectroscopy after reaction (Figures S19–S21, Supporting Information). Five aryl sulfide substrates with different electron‐donating and electron‐withdrawing ability functional groups have been tested to study the scalability of the proposed TPPS‐BV photocatalyst (**Figure** **7** and **Table** **1**). The photocatalytic yield decreased when increasing the electron‐withdrawing ability of the substituent group. This phenomenon can be attributed to the decrease of the electron density around the sulfur atom, induced by the electron‐withdrawing effect, which makes substrate more difficult to be oxidized.(**Scheme** **1**)

**Table 1:** Photocatalytic oxidation of sulfide with various substitutes using TPPS‐BV.^a)^

Entry	Substrate	Product	Conversion [%]	Selectivity [%]
1	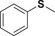	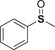	>99	>99
2	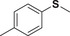	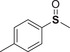	>99	>99
3	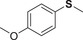	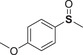	>99	>99
4	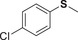	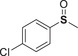	80	>99
5	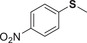	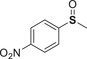	0	0

^a)^ Reaction conditions: 10 mg TPPS‐BV powder and 50 mm sulfide substrate in 2 mL MeOH, with magnetic stirring and an O_2_ balloon, irradiated with two 17 W LED lamp (420–750 nm), room temperature, 1 h.

**Figure 8:**
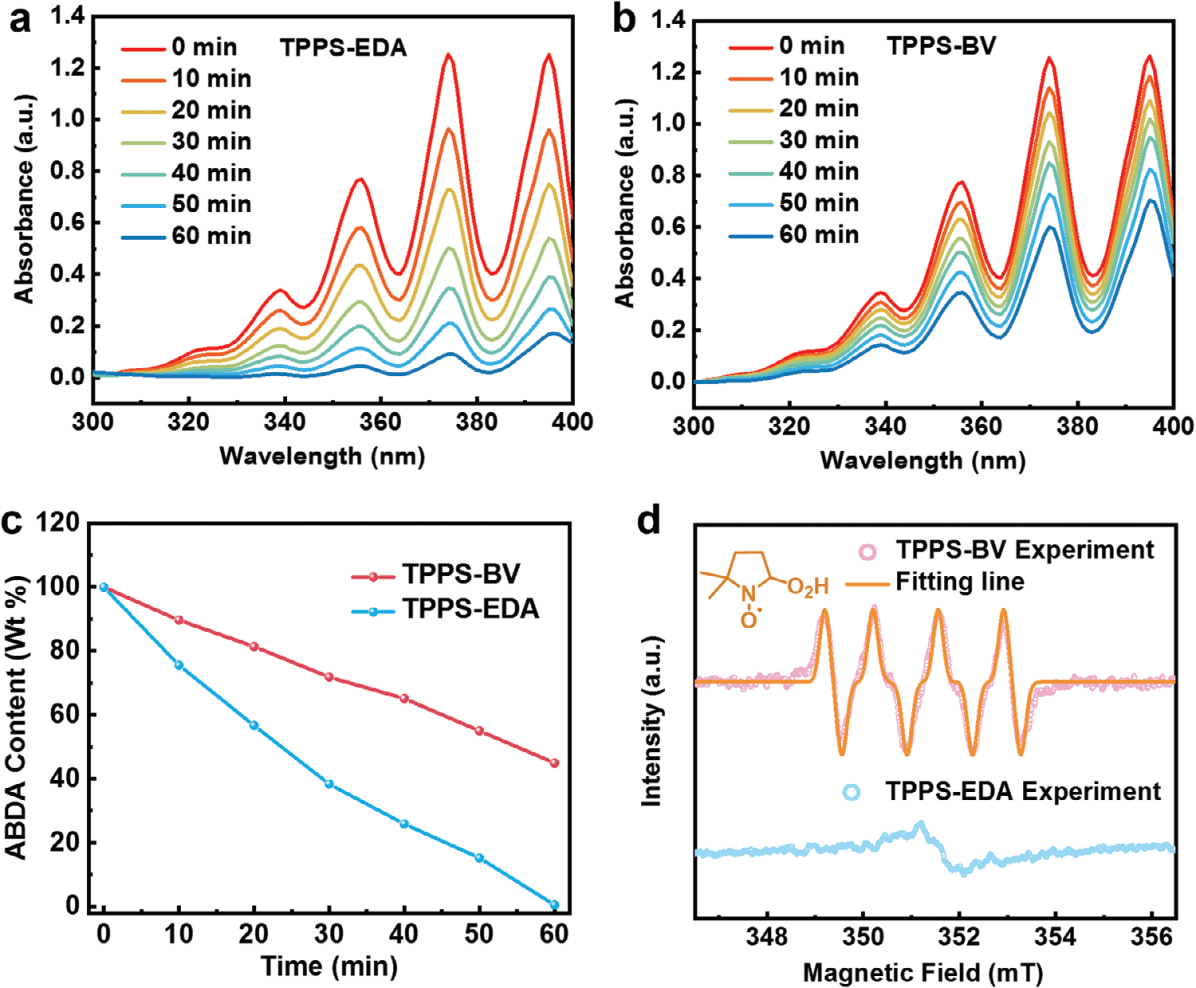
a,b) UV–vis absorption spectra of the ABDA under varied time illumination with TPPS‐EDA and TPPS‐BV, respectively; c) Absorbance evolution of ABDA solution at 355 nm after illuminating different time with TPPS‐BV or TPPS‐EDA; d) EPR spectrum of the resulting spin adduct DMPO•‐O_2_H (circles) and its corresponding EasySpin fitted spectrum (solid).

**Figure 9:**
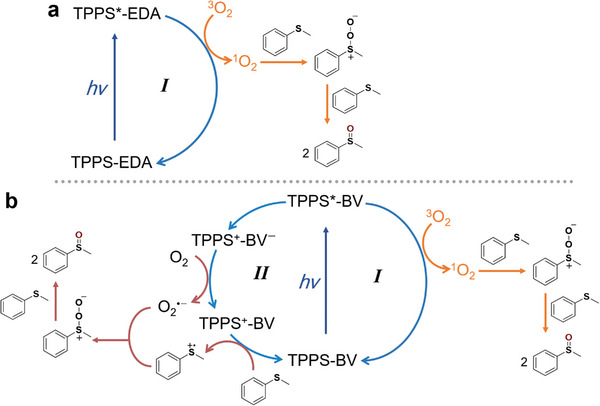
The proposed sulfide photooxidation mechanism from different photocatalytic systems, TPPS‐EDA and TPPS‐BV (thioanisole is used as a representative substrate here).

As it reported, there are two paths for the O_2_‐assisted sulfide oxidation reaction: singlet‐state oxygen (^1^O_2_) path and superoxide (O_2_^•−^) path.^[^ ^25^ ^]^ In the exploration of the impact of ^1^O_2_, a widely used probe for singlet oxygen, 9,10‐Anthracenediyl‐bis(methylene)dimalonic acid (ABDA), was employed. This study involved exposing ABDA solutions to TPPS‐EDA or TPPS‐BV, followed by illumination after purging with oxygen. The evolution of the UV–vis absorbance at 355 nm, which corresponds to ABDA's characteristic absorption peak, was then tracked (**Figure** **8**a,b).^[^ ^26^ ^]^ It was found that TPPS‐EDA exhibited a slightly faster absorption decline at 355 nm, indicating a faster generation of the ^1^O_2_ in TPPS‐EDA system (Figure 8c). Regarding the superoxide O_2_^•−^ path, electron paramagnetic resonance (EPR) experiments were performed using 5,5‐Dimethyl‐1‐pyrroline N‐oxide (DMPO) as the spin trap for O_2_^•−^. The recorded EPR spectrum showed a typical DMPO‐superoxide spin adduct, identified as DMPO^•^‐O_2_H, which is confirmed by EPR simulation and data fitting in the TPPS‐BV system (Figure 8d).^[^ ^27^ ^]^ However, under the same conditions, no discernible signal corresponding to DMPO^•^‐O_2_H spin adduct was observed in the TPPS‐EDA system, which also suggests the poor O_2_^•−^ production ability of the TPPS moiety in TPPS‐EDA system and TPPS‐EDA should follow the mechanism of path *I* (**Figure** **9**a). This outcome indicates that a notably higher capacity for O_2_^•−^ generation in the TPPS‐BV system compared to the TPPS‐EDA system should come from the reduced BV moiety.^[^ ^19a^ ^]^ To elucidate which one is the initial step: whether the electron transfer to BV or hole transfer to sulfide upon the TPPS being excited in this additional superoxide O_2_^•−^ path (path *II* in Figure 9b), variations in the photoluminescence (PL) lifetime of TPPS were studied using TCSPC measurements under ambient conditions, with different quenchers introduced. Notably, the PL life time of TPPS displays negligible change upon thioanisole introduction (11.2 to 11.4 ns), hinting at a lack of significant interaction between the excited TPPS and thioanisole substrate. In comparison, TPPS PL lifetime is diminished to 4.8 ns by the addition of BV, disclosing the occurrence of electron transfer from the excited TPPS to BV even in the presence of O_2_ (Figure S22, Supporting Information). This observation indicates that electron transfer from the excited TPPS to BV takes place in the initial step when both BV and thioanisole are concurrently present.

## 3 Conclusion

In conclusion, we have successfully designed and synthesized two distinct porphyrin‐based self‐assemblies TPPS‐EDA and TPPS‐BV by electrostatic forces. When employed as a heterogeneous photocatalyst for aryl sulfide oxidation, the TPPS‐BV assembly has demonstrated a remarkable four‐fold increase in catalytic efficiency compared to TPPS‐EDA. TPPS‐BV renders near 100% yield and selectivity of sulfoxide product of several substrates. Through a comprehensive investigation involving time‐correlated single photon counting (TCSPC), steady‐state photoluminescence (PL) quenching experiments, and the observation of characteristic absorption peaks of reduced‐state viologen, we have elucidated the photoinduced charge separation process between TPPS and BV. Mechanistic insights have revealed that while TPPS‐EDA facilitates aryl sulfide oxidation only through the singlet state oxygen path, TPPS‐BV introduces an additional path by generating superoxide due to its inherent photoinduced charge separation capability. Substrate scalability tests suggest that a high conversion efficiency can be achieved on aryl sulfides with electron‐donating units, while aryl sulfides with electron‐withdrawing ability make the reaction less favorable. Moreover, the satisfactory recyclability of TPPS‐BV has also been highlighted, maintaining consistent catalytic performance over four cycles of experiments. This study underlines the advantages of donor‐acceptor assemblies formed via electrostatic forces, suggesting their potential as promising photocatalysts for diverse photocatalytic applications beyond aryl sulfide oxidation.

## Conflict of Interest

The authors declare no conflict of interest.

## Supporting information

Supporting Information

## Data Availability

The data that support the findings of this study are available from the corresponding author upon reasonable request.
