# Supplementary material for: Recyclable and Stable Porphyrin‐Based Self‐Assemblies by Electrostatic Force for Efficient Photocatalytic Organic Transformation
Source: Adv Sci (Weinh). 2024 Mar 9;11(21):2308469. doi: 10.1002/advs.202308469 (PMC11151068; doi:10.1002/advs.202308469)
Supplement: Supplementary file 1 — Supporting Information [file ADVS-11-2308469-s001.pdf]

ADVANCED SCIENCE
Open Access

Supporting Information

for *Adv. Sci.*, DOI 10.1002/advs.202308469

Recyclable and Stable Porphyrin-Based Self-Assemblies by Electrostatic Force for Efficient Photocatalytic Organic Transformation

*Bin Cai, Ping Huang, Yuan Fang and Haining Tian**

Supporting Information

Recyclable and Stable Porphyrin-based Self-assemblies by Electrostatic Force for Efficient Photocatalytic Organic Transformation

Bin Cai[a], Ping Huang[a], Yuan Fang[b] and Haining Tian[a]*

a. Dr. B. Cai, Dr. P. Huang, and Prof. Dr. H. Tian
Department of Chemistry-Ångström Lab.
Uppsala University
Box 523, SE 751 20, Uppsala, Sweden
b. Dr. Y. Fang
Department of Chemistry
KTH Royal Institute of Technology
Teknikringen 30–36, 100 44 Stockholm, Sweden
E-mail: haining.tian@kemi.uu.

1. General Information

Meso-tetra (4-sulfonate phenyl) porphyrin (TPPS) was from TCI. Benzyl viologen (BV), ethylenediamine, methanol, 9,10-Anthracenediyl-bis(methylene) di malonic acid (ABDA), diethyl ether, tetrabutylammonium hexafluorophosphate ($TBAPF_6$), N, N-Dimethylformamide (DMF) and hydrochloric acid was from Sigma-Aldrich. All the chemicals were directly used without further purification. UV-vis absorption spectroscopy was measured with Shimadzu UV-1900i; Steady-state and time-related PL spectroscopy were measured with Edinburgh spectrofluorometer FS5. Scan electron microscopy (SEM) was measured with SEM – Zeiss LEO 1530 with Oxford AZtec EDS system. ^{1}H-NMR was measured with JEOL 400Y. FT-IR spectrum was obtained from Bruker VERTEX 70V FT-IR Spectrometer. XRD was measured in Bruker D8 Powder. Electrochemistry was measured in Autolab Nova, Metrohm. Gas chromatography-mass spectroscopy was obtained from Agilent 5977B GC/MSD instrument.

2. Ethylenediammonium dichloride synthesis

3 g ethylenediamine (0.05 mol) was added into 50 mL diethyl ether in an ice-water bath, and 10 mL 10M HCl was slowly added under stirring. The produced white powder precipitate was then filtered, and washed with a large amount of diethyl ether. The obtained powder was dried under a vacuum and used without further purification. ^{1}H-NMR (400 MHz, d-DMSO) δ 8.49 (6H, s), 3.09 (4H, s)

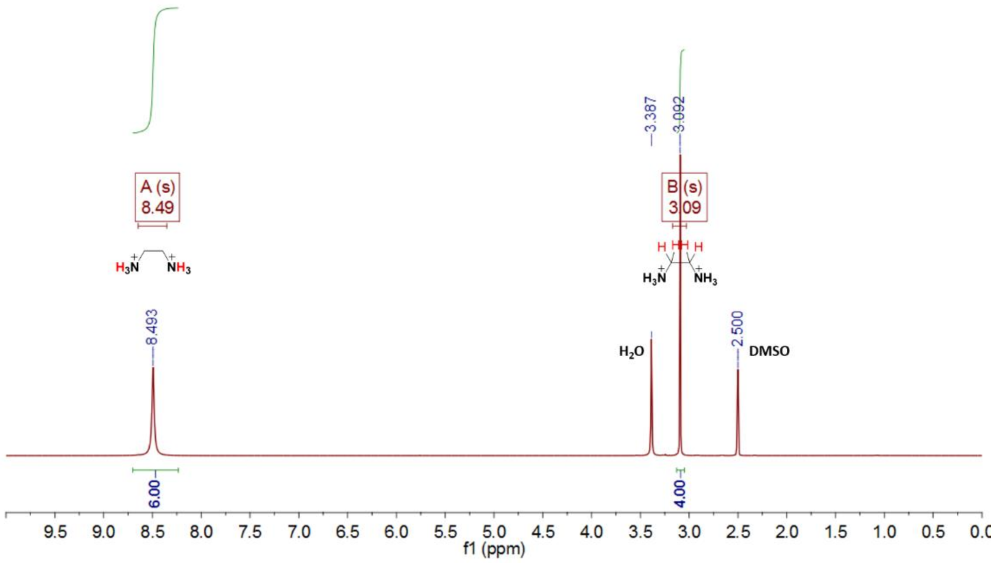

Figure S 1. ^{1}H-NMR spectrum of the synthesized EDA•2Cl

3. TPPS-BV and TPPS-EDA self-assemblies preparation scheme

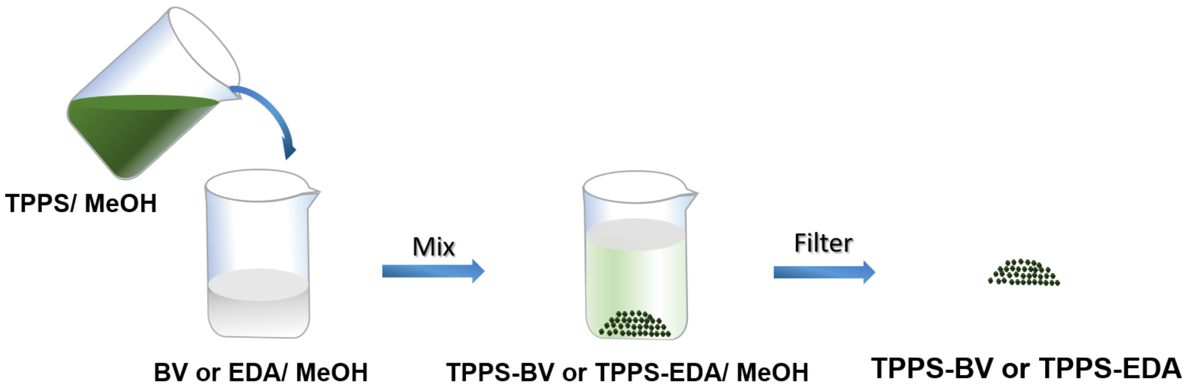

Figure S 2. TPPS-BV and TPPS-EDA self-assemblies preparation procedure.

Normally, 470 mg TPPS dissolved in methanol was slowly poured into 410 mg BV or EDA in 1L MeOH. Then the green precipitate instant appeared and dried under vacuum after filtration and washing.

4. Scanning electron microscope image of the TPPS-BV and TPPS-EDA

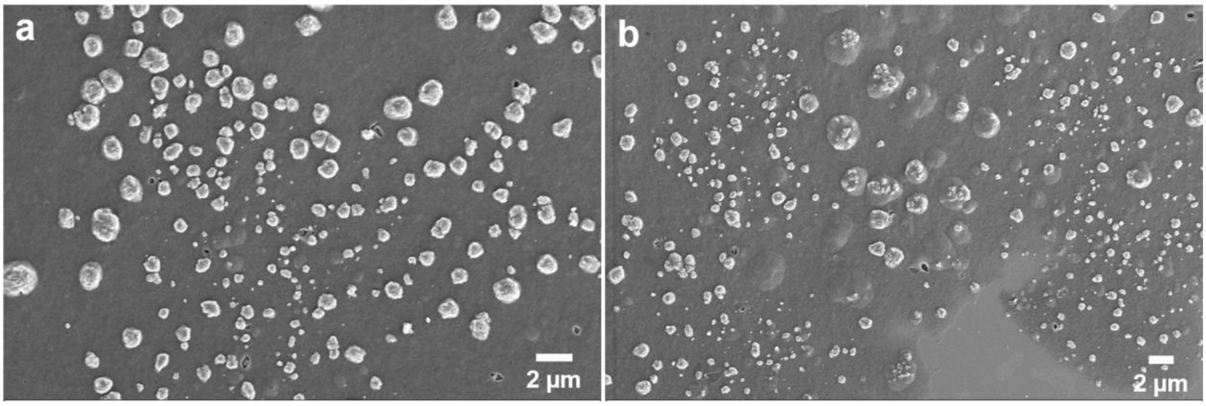

Figure S 3. SEM image of the (a) TPPS-BV self-assembly; (b) TPPS-EDA self-assembly

The SEM image prepared under a relatively lower concentration reveals that the TPPS-BV and TPPS-EDA consist of small nanoparticles with a size less than less than 1 μm.

5. Solid-state CP/MAS ^{13}C NMR Measurements

The CP/MAS ^{13}C-NMR measurements were performed with a Bruker 500 Avance HD spectrometer at the resonance frequency of carbon of 125.721 MHz. The probe used is a 4-mm double-resonance magic-angle spinning (MAS) probe. The conventional {1H-13C} CP experiments were used for high-resolution 13C measurements. 4 mm rotors were tightly packed with samples and their spectra were recorded at 8 kHz spinning frequency. Each sample contains around 80-90 mg. The 13C $\pi/2$ pulse length is 4.5 μs. All signal was recorded under dipolar decoupling. The contact time was set to 1 ms. A total of 128-2048 transients were collected for each spectrum with a recycle delay of 5 s. 13C signal of Adamantane was used as an external reference for chemical shifts (the signal at the lower field is set to 38.5 ppm). All the measurements were conducted at 298 K.

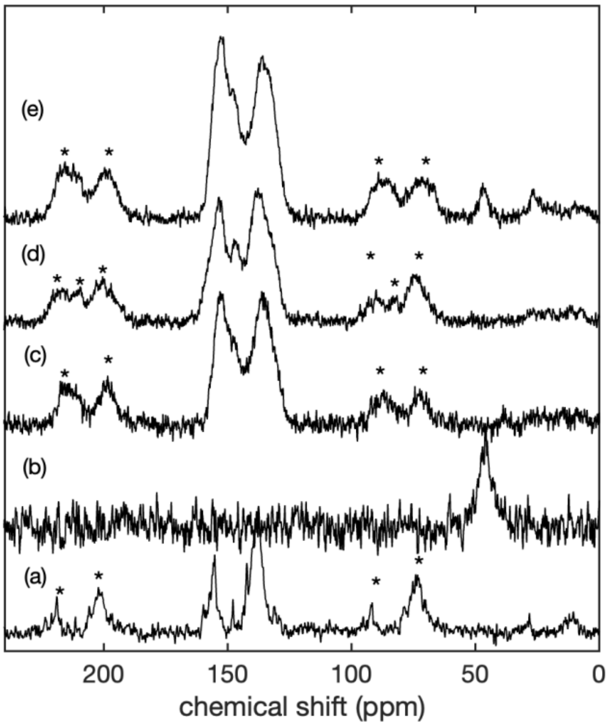

Figure S 4. The ^{13}C CP/MAS spectra of (a) BV (b) EDA (c) TPPS (d) TPPS-BV (e) TPPS-EDA. The spinning sidebands are marked with asterisks *.

The solid-state ^{13}C NMR spectra of TPPS-BV in the aromatic ring region exhibit broader signals compared to those of individual TPPS, likely due to the spectral overlap with the BV. Additionally, the peak around 45 ppm in the ^{13}C NMR spectra of TPPS-EDA should be attributed to the EDA. The relatively broad of peaks in the solid-state NMR spectrum are the characteristic features of amorphous structures, aligning with findings from XRD measurements.

6. Cyclic voltammetry of the TPPS and BV measured in DMF

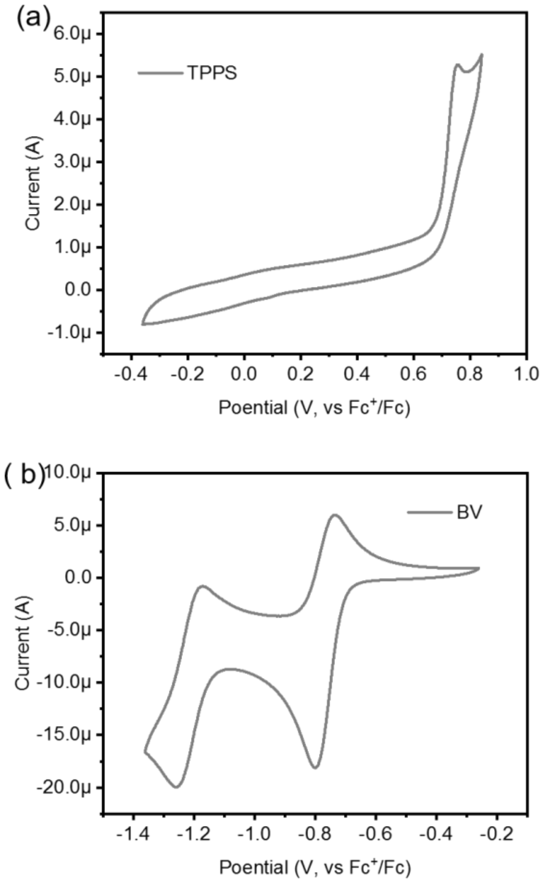

Figure S 5. Cyclic voltammetry (CV) of the TPPS and BV measured in DMF, with 0.1 M $TBAPF_6$ as the supporting electrolyte, $Ag/AgNO_3$ as the reference electrode, Pt wire as the counter electrode, glassy carbon as the working electrode, scan rate of 50 mV s^{-1}.

All redox potentials are relative to the (standard hydrogen electrode) SHE, with a formula as follows:

$$E\ (\text{vs SHE}) = E\ (\text{vs } Ag/AgNO_3) - E\ (\text{Fc vs } Ag/AgNO_3) + E\ (\text{Fc vs SHE})$$

Here, in the right equation, the first part is obtained from the measured CV value in DMF, the second part is obtained from directly adding ferrocene into the solution as the external reference after measuring the sample in DMF, the third part E(Fc vs SHE) = 0.63 V when measured in DMF, which obtained from the reference.[1]

Table S 1. Redox potentials of the respective TPPS and BV vs. SHE.

$E_{TPPS^+/\mathrm{TPPS}}$	$E_{0\text{-}0}$	$E_{TPPS^+/\mathrm{TPPS}^*}$	ΔE_{st}	$E_{TPPS^+/\mathrm{TPPS}^{3*}}$	E_{BV^{2+}/BV^+}
1.28 V	1.92 V	-0.64 V	0.33 V	-0.31 V	-0.21 V

The optical band gap $E_{0\text{-}0}$ of the TPPS was calculated according to the following formula:

$$E_{0-0} = \frac{1240}{\lambda_{intersect}}$$

Where the $\lambda_{intersect}$ is the wavelength at the intersecting point of the normalized TPPS UV-vis absorption spectrum and normalized TPPS PL emission spectrum.

$E_{TPPS^+/\mathrm{TPPS}^*}$ is the oxidation potential of the singlet-state excited TPPS, which is calculated from: $E_{TPPS^+/\mathrm{TPPS}^*}$=$E_{TPPS^+/TPPS} - E_{0\text{-}0}$;

ΔE_{st} is the energy gap (ΔE_{ST}) between S_1 and T_1 state of the porphyrin,which has been reported around 0.33 eV;[2]

$E_{TPPS^+/\mathrm{TPPS}^{3*}}$ is the oxidation potential of the triplet-state excited TPPS, which is calculated from: $E_{TPPS^+/\mathrm{TPPS}^{3*}}$=$E_{TPPS^+/\mathrm{TPPS}^*} + \Delta E_{st}$;

E_{BV^{2+}/BV^+} is the reduction poential of the BV, which is obtained from the CV measurements."

7. UV-vis absorption spectra of the TPPS-BV and TPPS-EDA self-assemblies

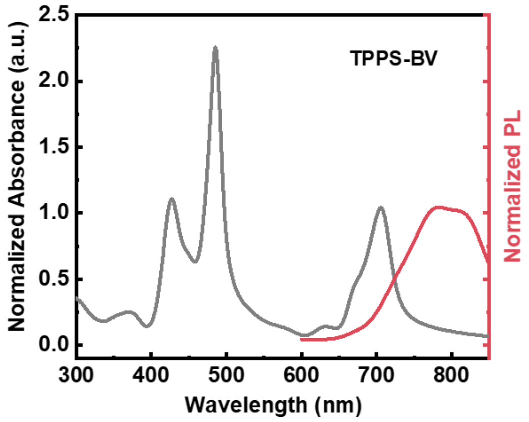

Figure S 6. UV-vis absorption spectrum and PL emission spectrum of the TPPS-BV.

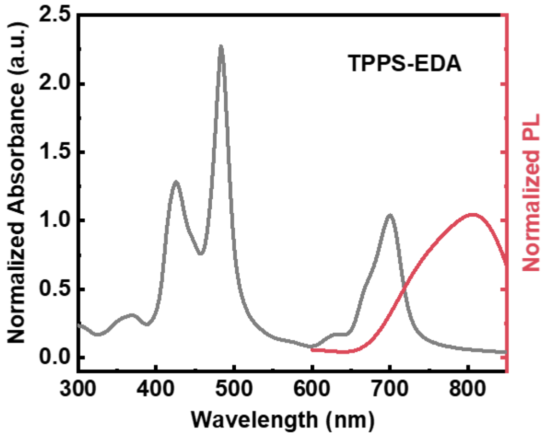

Figure S 7. UV-vis absorption spectrum and PL emission spectrum of the TPPS-EDA.

The UV-vis absorption spectra of TPPS-BV and TPPS-EDA were recorded in MeOH after 2 hours of sonication, ensuring complete dispersion. The side-absorption peaks at 483 nm for TPPS-EDA and 486 nm for TPPS-BV should belong to molecular aggregations. Furthermore, in the solid state, both TPPS-BV and TPPS-EDA self-assemblies exhibit enhanced absorption around 700 nm compared to that measured in the DMSO solution. Additionally, there is a red shift in the absorption peak from 698 nm in TPPS-EDA to 707 nm in TPPS-BV. This shift could be attributed to a stronger aggregation in the TPPS-BV self-assembly or the formation of a charge transfer complex within the TPPS-BV self-assembly. In addition, the normalized PL emission spectra of the TPPS-BV and TPPS-EDA measured in the film state have also been provided.

8. Cyclic voltammetry spectroscopy of the TPPS-BV and TPPS-EDA

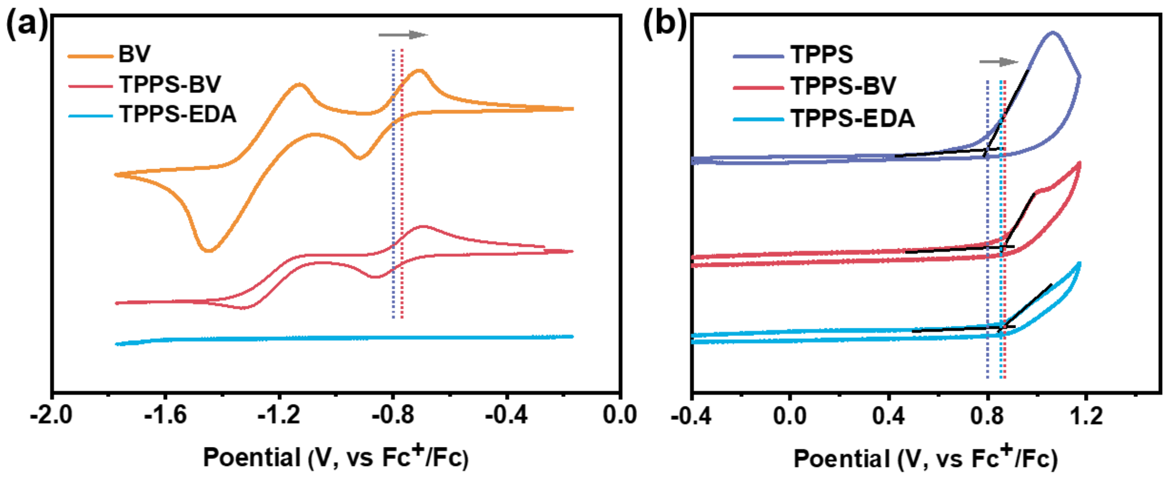

Figure S 8. Cyclic voltammetry spectroscopy of the BV film, TPPS film, TPPS-EDA film, and TPPS-BV film (a) reduction part; (b) oxidation part. With Ag/$AgNO_3$ as the reference electrode, Pt wire as the counter electrode, 0.1 M $TBAPF_6$ in DCM as the electrolyte.

TPPS-BV or TPPS-EDA first mixed with 5 wt% Nafion/ EtOH solution and EtOH (with a ratio of self-assembly: Nafion: EtOH = 0.1 mg: 10 μL: 200 μL), then drop-casting onto the glassy carbon working electrode. The cyclic voltammetry measurements of the as-prepared TPPS-BV and TPPS-EDA self-assemblies were then performed. For a comparison, the reduction region of the individual BV film, and oxidation region of the individual TPPS film were also measured. It can be found that the reduction potential of the BV moiety and oxidation potential of the TPPS moiety in the TPPS-BV self-assembly both slightly positive-shifted compared to the individual BV and TPPS. No reduction peak was detected in the measured region for TPPS-EDA. Moreover, the oxidation potential of the TPPS-EDA is close to that of the TPPS-BV, as shown in Figure S8.

9. UV-vis absorption spectroscopy of the TPPS in DMSO solution

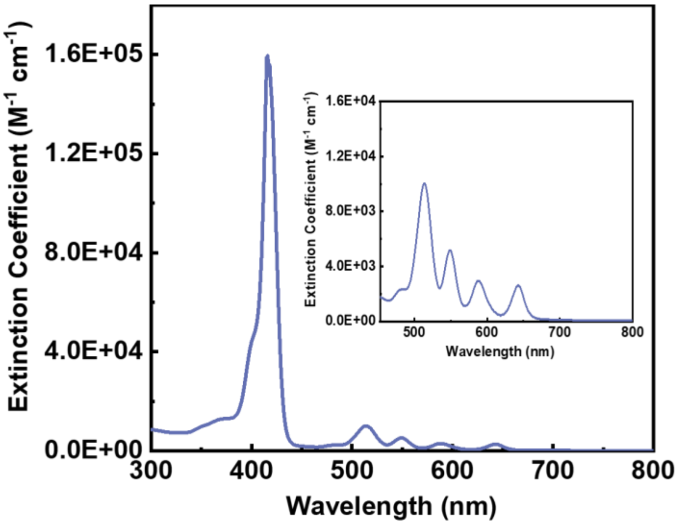

Figure S 9. UV-vis absorption spectroscopy of the TPPS measured in DMSO inserted small graph is the magnified Q band.

11. TCSPC measurements of the TPPS quenched with BV or TEOA

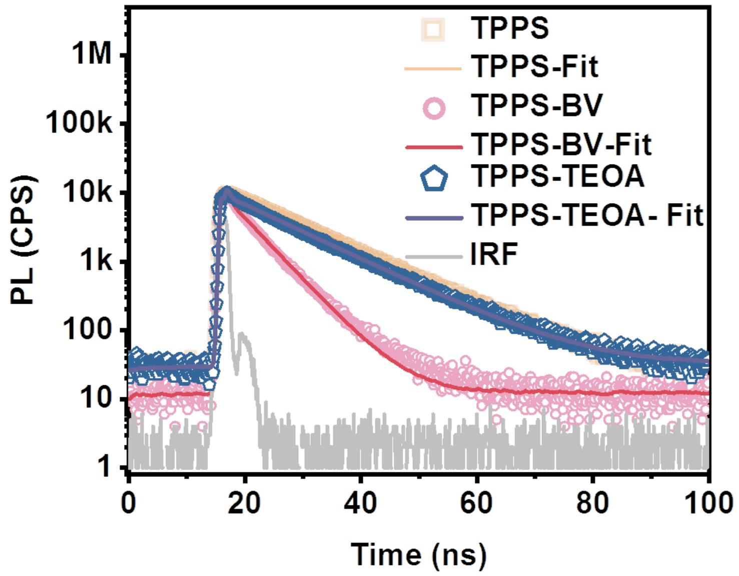

Figure S 10. TCSPC measurements of TPPS (yellow square), TPPS with 0.8 mM BV (pink circle), TPPS with 0.8 mM TEOA (blue pentagram), and the solid lines are corresponding fitting curves.

Variations in the photoluminescence (PL) lifetime of TPPS were studied using TCSPC measurements under ambient conditions, with different quenchers introduced. Specifically, the PL lifetime of TPPS displayed negligible change upon TEOA introduction (11.2 ns to 10.8 ns), hinting at a lack of significant interaction between the excited TPPS and TEOA. In comparison, TPPS PL lifetime was diminished to 4.8 ns by the addition of BV, disclosing the occurrence of electron transfer from the excited TPPS to BV should happen in the first step.

12. TCSPC Fitting parameters

The fitting lines were fitted with the following formula by subtracting the instrument response function (IRF):

$$\mathrm{y} = \mathrm{y}_0 + \mathrm{A}_1 e^{-(x-x_1)/\tau_1} + \mathrm{A}_2 e^{-(x-x_1)/\tau_2}$$

$$t = \frac{A_1{\tau_1}^2 + A_2{\tau_2}^2}{A_1\tau_1 + A_2\tau_2}$$

Table S 2. TCSPC fitting parameters of the TPPS PL lifetime by different quenchers

	TPPS	TPPS+BV	TPPS+EDA	TPPS+thioanisole	TPPS+TEOA
A_1	0.17	0.1	0.17	0.17	0.26
τ_1 (ns)	0.04	0.5	0.05	0.1	0.2
A_2	0.83	0.9	0.83	0.83	0.74
τ_2 (ns)	11.2	4.8	11.4	11.4	10.9
t (ns)	11.2	4.8	11.4	11.4	10.8

13. Reaction Setup

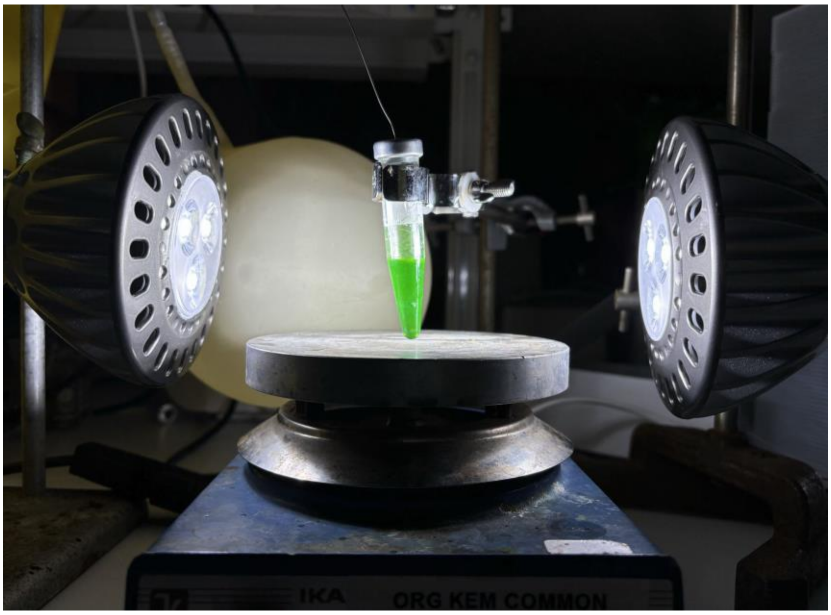

Figure S 11. Photocatalytic sulfide oxidation setup

The photocatalytic reaction was executed employing two Zenaro brand LED lamps (17W, emitting spectrum: 420 to 750 nm), with each lamp an intensity of 50 mW cm^{-2}. The reaction mixture consisted of 10 mg of TPPS-BV powder and 50 mM sulfide substrate in 2 mL MeOH, subjected to magnetic stirring. The reaction vessel was equipped with an oxygen balloon and was conducted at room temperature.

A 100 µL aliquot was taken from the reaction vial at specific time intervals. Subsequently, 900 µL of dichloromethane (DCM) was added to dilute the sample. Anisole was employed as the internal standard to facilitate the quantification of converted thioanisole and generated methyl phenyl sulfoxide. The resulting diluted sample was introduced into the GC-MS system using an automated sample injection setup (Agilent 5977B GC/MSD instrument with argon as the carrier gas). To provide reference points, pure thioanisole (the initial reactant) and methyl phenyl sulfoxide (the product) were also injected into the GC to identify their respective retention times. Conversion and selectivity efficiencies were calculated based on GC peak area integration, utilizing the following equations:

$$\text{conversion}(\%) = \frac{n_{initial\ thioanisole} - n_{residual\ thioanisole}}{n_{initial\ thioanisole}} \times 100\%$$

$$\text{Selectivity}(\%) = \frac{n_{methyl\ phenyl\ sulfoxide}}{n_{initial\ thioanisole} - n_{residual\ thioanisole}} \times 100\%$$

14. Comparison of the photocatalytic activity of the homogeneous and heterogeneous system

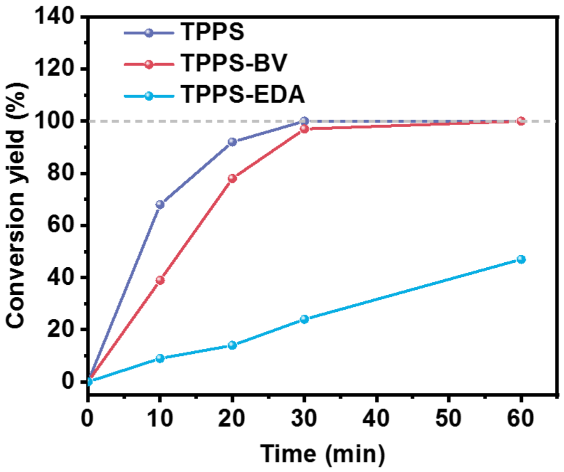

Figure S 12. Photo-oxidation of thioanisole under different conditions: "light+O_2+TPPS-BV" (red line), "light+O_2+TPPS-EDA" (blue line). 2mL 50 mM thioanisole added with 10 mg photocatalyst in the presence of O_2.

15. Picture of reaction solutions with TPPS, TPPS-BV and TPPS-EDA

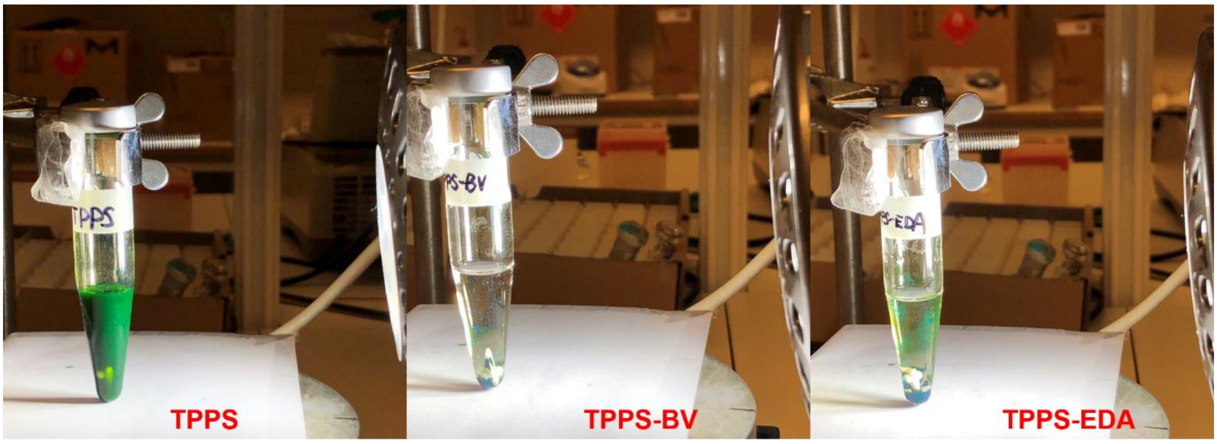

Figure S 13. Reaction pictures of the homogenous TPPS and heterogeneous TPPS-BV as well as TPPS-EDA.

It can be found that TPPS can be dissolved in a methanol solvent and act as a homogeneous catalyst. Both TPPS-BV and TPPS-EDA cannot be dissolved in methanol solvent, thus acting as heterogeneous catalysts.

16. Quantum efficiency measurements based on TPPS-BV

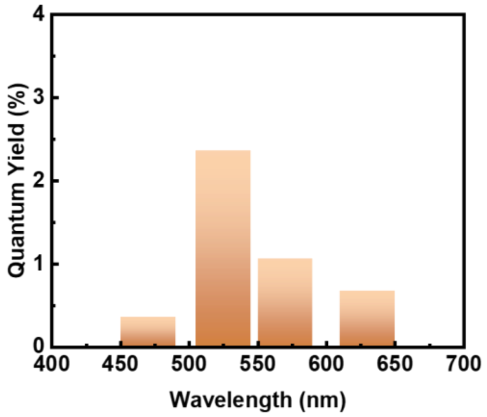

Figure S 14. Quantum efficiency of the photocatalytic thioanisole oxidation reaction based on TPPS-BV.

The quantum efficiency was defined as the ratio between the number of carriers involved in the reaction and the number of all the incident photos on the active area at a given wavelength. It could be calculated according to the following equation:

$$QY\% = \frac{{}^{1}/_{2} \times n_{methyl\ phenyl\ sulfoxide} \times N_A \times h \times c}{I \times t \times A \times \lambda}$$

Where $n_{methyl\ phenyl\ sulfoxide}$ is mole of the photogenerated product, N_A is the Avogadro´s constant (6.02*10^{23} mol^{-1}), h is the Planck constant (6.60*10^{-34} Js^{-1}), c is the speed of light (3*108 ms-1), I is the light intensity, λ is the wavelength of the incident monochromatic light, A is the irradiation area, t is the irradiation time.

17. Different ratio of the TPPS to BV (or EDA) study

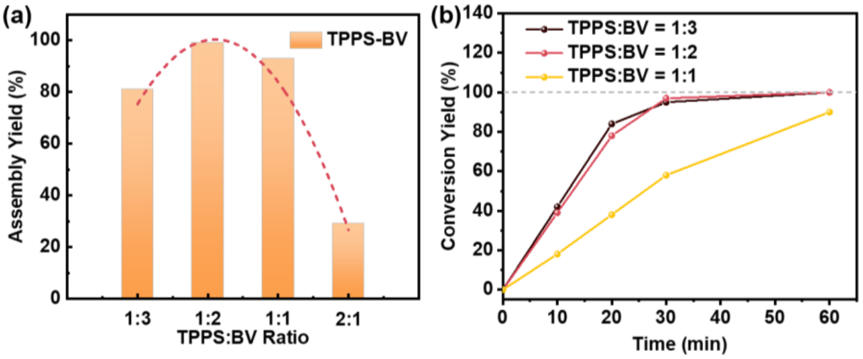

Figure S 15. (a) TPPS-BV self-assembly yield, (b) Photocatalytic sulfide oxidation activity against different TPPS and BV ratios.

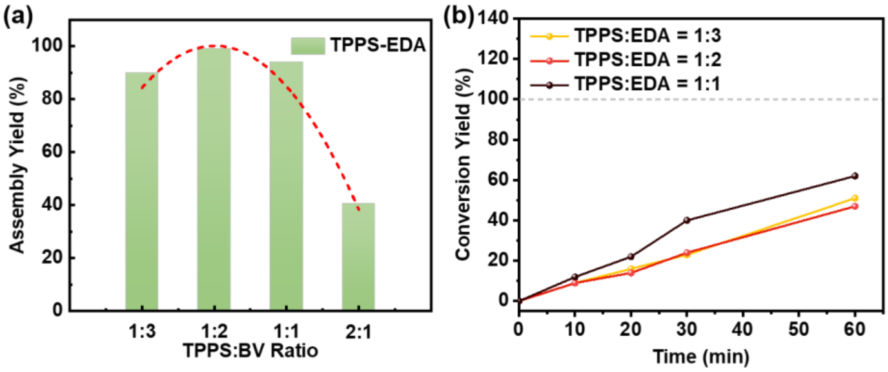

Figure S 16. (a) TPPS-EDA self-assembly yield, (b) Photocatalytic sulfide oxidation activity against different TPPS and EDA ratios.

Different molar ratios of the TPPS to BV or EDA have been conducted to fabricate the TPPS-EDA and TPPS-BV self-assemblies and to compare their photocatalytic performance. Given that the TPPS contains four anion groups and BV (or EDA) only possesses two cation groups, it is found that when TPPS: BV (or EDA) = 1:2 is the optimal condition considering that the TPPS-BV (or EDA) self-assembly's yield (the mass ratio of obtained self-assembly to the sum of monomers) is highest under this ratio condition (With the ratio of 1:1 will lose some TPPS, while the ratio of 1:3 will lose some BV or EDA). To ensure the self-assembly yield, only TPPS: BV (or EDA) = 1:1, 1:2, and 1:3 have been chosen to perform the photocatalytic reaction. Moreover, no obvious improvement of the thioanisole oxidation rate could be observed when slightly increasing the cation ratio (BV or EDA) from 1:2 to 1: 3. While an obvious decrease of the thioanisole oxidation rate could be observed when decreasing the cation ratio (BV) in TPPS-BV from 1:2 to 1:1. On the other hand, a slightly improvement of the thioanisole oxidation rate has been observed when decreasing the cation ratio (EDA) in TPPS-EDA from 1:2 to 1:1, which might be caused by the slightly improved ratio of the photoactive TPPS in the TPPS-EDA self-assembly.

18. Thioanisole photooxidation with TIPS-BV as the catalyst for a relatively long time

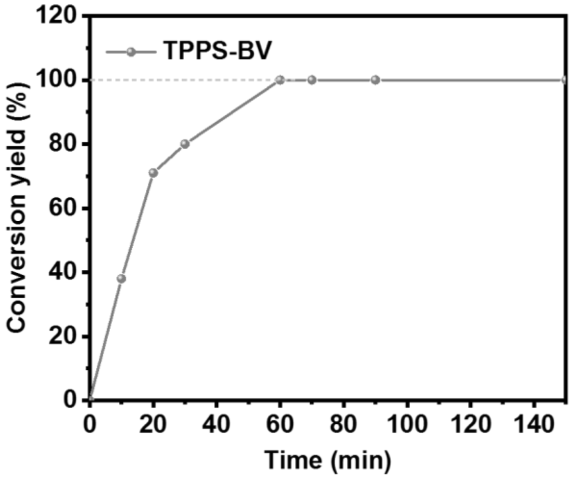

Figure S 17. Thioanisole photooxidation with TPPS-BV as the catalyst. 2mL 50mM thioanisole in MeOH, 10 mg TPPS-BV under O_2 atmosphere, two LED lamps with each 50 mW cm^{-2} LED light (420-750 nm).

19. TPPS-BV recycle experiment

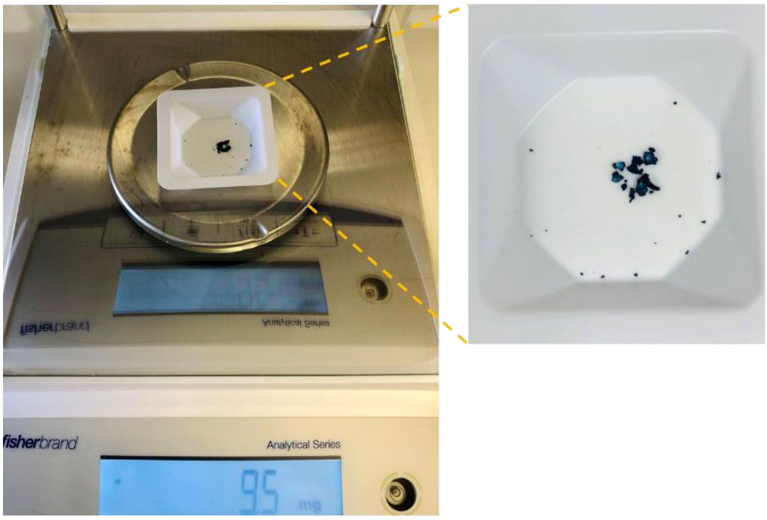

Figure S 18. TPPS-BV powder was recycled from the first round of the experiment after washing and drying. The initially loaded amount is 10 mg, and 9.5 mg can be recycled.

20. Catalyst stability after the photocatalytic reaction

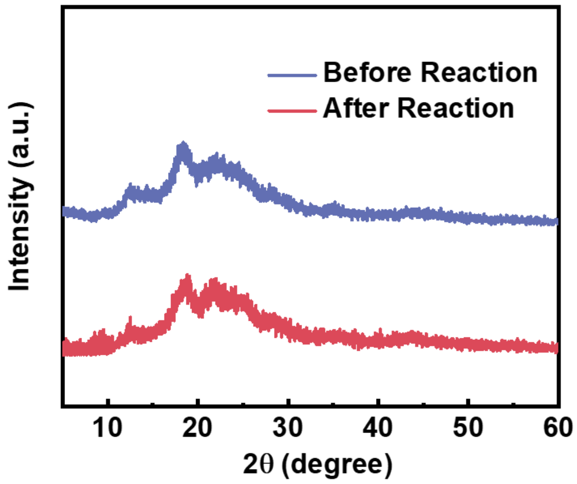

Figure S 19. Powder XRD spectroscopy comparison of the TPPS-BV before and after the photocatalytic reaction.

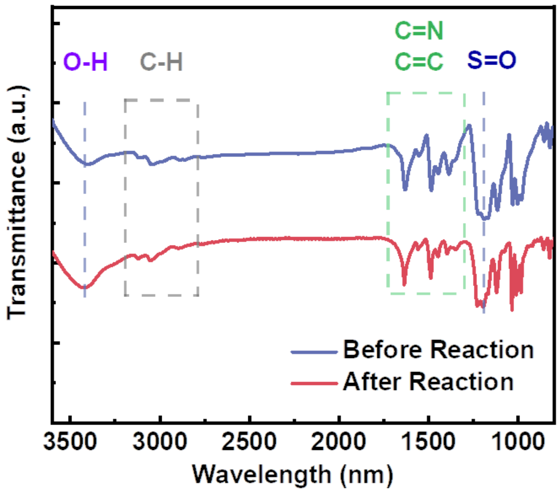

Figure S 20. FT-IR spectroscopy comparison of the TPPS-BV before and after the photocatalytic reaction.

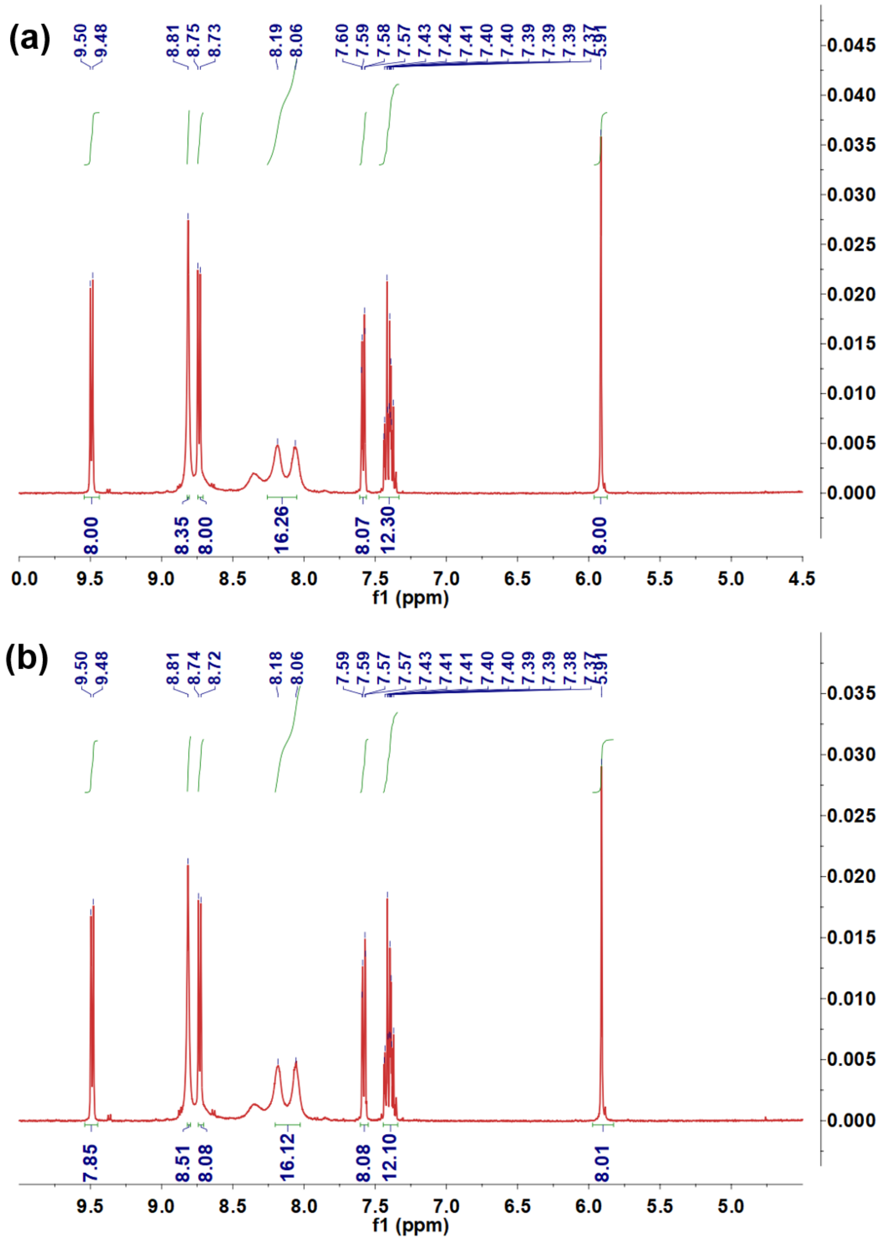

Figure S 21. ^{1}H NMR spectroscopy in d-DMSO comparison of the TPPS-BV (a) before and (b) after the photocatalytic reaction.

21. Determination of the initial photo-induced charge transfer step in route III

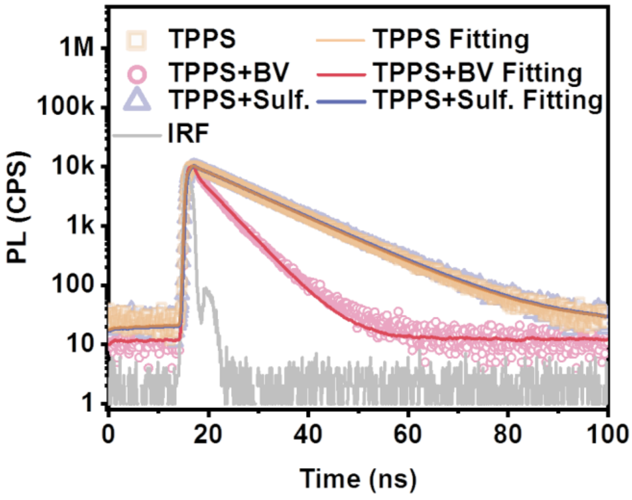

Figure S 22. TCSPC measurements of TPPS (yellow square), TPPS with 0.8 mM BV (pink circle), TPPS with 0.8 mM Thioanisole (purple triangle), and the solid lines are corresponding fitting curves

22. Electron paramagnetic resonance measurement

Electron paramagnetic resonance (EPR) spectroscopy analysis was performed on a Bruker EMX-micro spectrometer equipped with an EMX-Primium bridge and an ER4119HS resonator and controlled with Bruker Xenon software. DMPO (5,5-Dimethyl-1-pyrroline-N-oxide, ≥ 97%, Sigma-Aldrich) was used as a spin trap for superoxide superoxide $O_2^{\bullet-}$ radicals.

2 mg mL^{-1} TPPS-BV or TPPS-EDA in MeOH was added with 0.1 M DMPO solution. The sample was loaded into a glass capillary (Hirschmann, Germany, diameter: 1.75 mm, 75 mm/100 µL), and illuminated for 3 minutes after purging with O_2 before the measurements. The spectral data fitting was carried out using EasySpin software packet (free version easy spin 6.0.0-dev.53) was used.[3] The fitted parameters are listed as follows:[4]

Parameters	g	α_N =38.1228 MHz	α_H(Beta)= 28.1488 MHz	α_H(OOH)=5.0000 MHz
Simulation	2.0061	1.3578 mT	1.0025 mT	0.1781 mT

Reference

[1] H. Tian, L. Sun, *Journal of Materials Chemistry* **2011**, 21, 10592.

[2] a) M. Wolf, D. Lungerich, S. Bauroth, M. Popp, B. Platzer, T. Clark, H. L. Anderson, N. Jux, D. M. Guldi, *Chemical Science* **2020**, 11, 7123; b) B. Fu, Y. Che, X. Yuan, L. Sun, H. Xu, J. Zhao, L. Liu, *Dyes and Pigments* **2021**, 196, 109754; c) D. M. Togashi, S. M. B. Costa, *Physical Chemistry Chemical Physics* **2002**, 4, 1141.

[3] a) S. Stoll, in *Multifrequency Electron Paramagnetic Resonance*, DOI: https://doi.org/10.1002/9783527672431.ch3 **2014**, p. 69; b) S. Stoll, A. Schweiger, *Journal of Magnetic Resonance* **2006**, 178, 42.

[4] a) C. Corvaja, in *Electron Paramagnetic Resonance*, DOI: https://doi.org/10.1002/9780470432235.ch1 **2009**, p. 1; b) K. Makino, T. Hagiwara, A. Murakami, *International Journal of Radiation Applications and Instrumentation. Part C. Radiation Physics and Chemistry* **1991**, 37, 657.
